# Radiology websites: Conventional radiology

**Published:** 2008-11

**Authors:** IK Indrajit

**Affiliations:** Department of Radiodiagnosis and Imaging, Army Hospital (Research and Referral), Delhi Cantt – 110 010, India

**Few useful websites related to conventional radiology are reviewed below:**

1. **The X-ray Century** at http://www.emory.edu/X-RAYS/century.htm is a set of absorbing articles with historical value relating to Roentgen's discovery of x-rays. The material has been created by Peter Sprawls PhD, from the Department of Radiology at Emory University. It is presented in the form of newsletters written with a ‘Back to the Future’ feel. Three chapters take a peek into the past by covering the ‘Crookes Tube,’ ‘Experiments with X-Rays,’ and ‘Fluoroscope and Other Apparatus.’ There are also sections covering the origins and early work on history of gas discharge tubes, Prof. Roentgen's discovery a new kind of ray, the series of investigations which led Dr Roentgen to write his first paper, Becquerel's discovery of radioactivity, etc.
2. **The History of Radiology** at http://medinfo.ufl.edu/other/histmed/klioze/ offers educative presentations on a variety of topics related to the evolution of radiology. The material is authored by Scott Klioze and produced by the Office of Medical Informatics, University of Florida. There are sections titled ‘Discovery of X-rays,’ ‘Wilhelm Conrad Roentgen,’ ‘Roentgen's Laboratory,’ ‘November 8, 1895, Bremsstrahlung’, ‘Modern Tube,’ ‘First Medical Radiograph,’ and ‘Angiography;’ in addition, there are features on Godfrey Hounsfield and Dr Paul Lauterbur. This presentation is best viewed with Real Player, which is available as a free download from www.real.com.
3. **The British Society for History of Radiology** at http://www.bshr.org.uk/index.html is the official website of an organization formed in January 2006. It has absorbing sections on the evolution of radiology equipment and the history of radiology, and images and movies of the practice of radiology in the early 20^th^century.
4. **Physical Principles of Medical Imaging** at http://www.sprawls.org/resources/ is a ‘comprehensive online textbook covering the physics and technology of the medical imaging modalities.’ Authored by Perry Sprawls, Professor Emeritus, Department of Radiology, Emory University School of Medicine, Atlanta, the textbook is an open resource available without charge and is periodically updated. The material covered includes radiation, radiography, mammography, fluoroscopy, computed tomography, ultrasound, radionuclide imaging, digital image systems, and magnetic resonance imaging. For each topic there are outlines, learning objectives, teaching visuals, online modules, and a text reference. A web-based edition offering the salient topics from the 2^nd^edition of the textbook ‘The Physical Principles of Medical Imaging’ is available at http://www.sprawls.org/ppmi2/.
5. **Medical Radiography** at http://home.earthlink.net/∼terrass/radiography/medradhome.html or http://web.wn.net/∼usr/ricter/web/medradhome.html is a radiography and radiophysics portal maintained by Richard Terrass of the Dept. of Radiology, Massachusetts General Hospital. The site features resources on physics, radiation protection, and radiologic anatomy and education resources. Besides, there are features on radiology history, general anatomy, and medical imaging and informatics. There is an admirable quick search tool that can be very useful.
6. **Nick's X-ray site** at http://www.e-radiography.net/ is a diagnostic radiography site with an impressive array of educative material. There are sections covering basic topics like history of x-rays and ImageBase. Radpathindex at http://www.e-radiography.net/radpath/radpathindex.htmbriefly analyses topics ranging from achalasia cardia to xanthogranuloma of bone. Tutorial notes at http://www.e-radiography.net/nickspdf/tutnotes.htm offer topics such as ‘Introduction to X-Ray Tubes,’ ‘Mobile Radiographic Equipment,’ ‘Intensifying Screens,’ ‘Introduction to Chest,’ ‘Extremity,’ ‘GI Tract,’ ‘Fluoroscopy,’ ‘Intravenous Urography,’ and ‘skull Radiography.’ The section titled ‘Rad Reports’ covers topics from abdomen to torus fractures of the radius and ulna. A ‘Radiographic Technology Index’ at http://www.e-radiography.net/radtechindex.htm offers brief material on radiophysics.
7. **ChestX-Ray.com** at http://www.chestx-ray.com/ is an educative website covering the gamut of chest radiology. The site, which is curated by Jud W. Gurney, has many incisive sections. In the ‘Research’ section, a neat interface is available which links to MEDLINE, thoracic journals, and statistical tools. The ‘Education’ section deals with lectures, tutorials, unknown cases, journal club, and teaching files. The ‘Practice’ section is an assorted compilation of protocols and standards in thoracic imaging. It includes guidelines for treatment of contrast reactions, American College of Radiology (ACR) standards and appropriateness criteria, CT protocols, guidelines for a radiology report, solitary pulmonary nodule (SPN) calculator, doubling time calculator, and an HRCT diagnosis tool.
8. **George Simon's X-Ray Collection** at http://myweb.lsbu.ac.uk/dirt/museum/g-topics.html is an educative site dealing with chest radiology. The images in the ‘George Simon Collection’ were ‘produced for the Museum of the Royal College of Radiologists in London, England’, and were originally sold as slides. Following permission of the Wardens of the College, the work has been edited for this medium by Ian Maddison, a Radiologist.' The pulmonary section of ‘X-ray Museum’ is available at http://myweb.lsbu.ac.uk/dirt/museum/chestpath.html. There are special educative web pages available on topics like obstructive atelectasis, normal and pathological hilar anatomy (specifically at http://myweb.lsbu.ac.uk/dirt/museum/gs-third.html), nodular shadows in the lungs, cardiac lesions, and congenital cardiac defects in neonates. Other teaching material in pulmonary radiology is available at http://myweb.lsbu.ac.uk/dirt/museum/topics.html#chest. Similarly, the cardiology section of ‘X-Ray Museum’ is available at http://myweb.lsbu.ac.uk/dirt/museum/heartpath.html.
9. **Radiology Board Review Notes** at http://radiology.creighton.edu/ is a site from Creighton University with comprehensive text material on different organ systems such as musculoskeletal, chest, gastrointestinal, genitourinary etc.; it also covers different modalities such as radiophysics, mammography, and nuclear medicine. Another feature of the website is the coverage on differential diagnosis. A ‘staging of Neoplasm’ handout, conveniently compiled in a single page is available at http://radiology.creighton.edu/staging.htm#. The website developed by James Brown et al. has additional educative sections such as ‘Resident Survival Guide,’ ‘Basic Imaging,’ ‘Interesting Cases,’ and ‘Case of the Week.’
10. **CHORUS** or **Collaborative Hypertext of Radiology** at http://chorus.rad.mcw.edu/ is available from the Medical College of Wisconsin and is edited by Charles E Kahn Jr. It is a ‘quick reference’ hypertext for physicians and medical students with more than 1,100 documents that describe diseases and radiological findings; it provides differential diagnosis lists (‘gamuts’); and relevant anatomy, pathology, and physiology. CHORUS is based on Fact/File, a radiology hypertext reference that has been used with a clinical radiology information system at the University of Chicago. This ‘quick reference’ hypertext is linked to ARRS Goldminer.

## End piece

**European Society of Radiology** is the principal forum for radiology education and practice in Europe. The web portal of ESR/ECR is available at http://www.myesr.org/cms/website.php which has many distinctive sections that cover ‘ESR/ECR News’ and ‘Abstract Submission for the Annual ECR Conference.’ A membership is essential to experience the various academic material on offer. Some of the e-learning tools on offer are eECR (a selection of scientific highlights of ECR), EPOS™ (Electronic Presentation Online System), EDIPS (ECR's Digital Preview System), and EURORAD. EURORAD at http://www.eurorad.org/ is a large peer-reviewed teaching database of medical information, imaging data, and radiological cases. It contains ‘case reports for medical students (simple cases), residents in radiology (everyday cases), and senior radiologists (complex cases).’ EURORAD has been enhanced with new features such as a fast search engine and a multilingual navigation tool that makes it a handy online learning reference in radiology.

**USS Radiology Education Gateway** at http://rad.usuhs.edu/ is an educative portal from the Department of Radiology, Uniformed Services University of the Health Sciences, Bethesda. The web page maintained by Chris Quarles is multimedia packed and has many distinctive sections. They include ‘Radiologic Differential Diagnosis,’ ‘Radiologic Anatomy,’ Distance Learning Lectures,’ ‘Electronic Handouts,’ Imaging Atlas,' and ‘Decision Support Tools.’ The neuroradiology resources include material on brain herniation, Brain Lesion Locator™, brain tumor - WHO classification, and brain vascular maps. The MedPix^®^ database inventory at http://rad.usuhs.mil/medpix/medpix.html?mode=default, currently has 43,390 images, 10,100 teaching file cases, and 6,244 peer-reviewed topics. Fashioned by J.G. Smirniotopoulos, and H. Irvine, the educative database can be searched by disease location, by organ system, by pathology category, by patient profiles, by image classification, and by caption. 

